# The integration of a Podiatrist into an orthopaedic department: a cost-consequences analysis

**DOI:** 10.1186/s13047-017-0227-0

**Published:** 2017-10-12

**Authors:** Tom P. Walsh, Linda R. Ferris, Nancy C. Cullen, Christopher H. Brown, Cathy J. Loughry, Nikki M. McCaffrey

**Affiliations:** 1 Department of Orthopaedics and Trauma, The Queen Elizabeth Hospital, Woodville South, South Australia Australia; 2 Department of Orthopaedics, Repatriation General Hospital, Daw Park, South Australia Australia; 3 Department of Podiatry, Central Adelaide Local Health Network, Adelaide, South Australia Australia; 4 Flinders Health Economics Group, Flinders University, Bedford Park, South Australia Australia; 5 Deakin Health Economics, Deakin University, Burwood, Victoria Australia

**Keywords:** Medical economics, Clinical pathways, Podiatry, Orthopaedics

## Abstract

**Background:**

The aim of this study was to evaluate the cost-consequences of a podiatry-led triage clinic provided in an orthopaedic department relative to usual care for non-urgent foot and ankle complaints in an Australian tertiary care hospital.

**Methods:**

All new, non-urgent foot and ankle patients seen in an outpatient orthopaedic department were included in this study. The patients seen between 2014 and 2015 by Orthopaedic Surgeons were considered ‘usual care’, the patients seen between 2015 and 2016 by a Podiatrist were considered the ‘Podiatry Triage Clinic’. Data on new and review patient appointments; the number of new patients / session; the number of appointments / patient; the number of patients discharged; the surgical conversion rate; staff time; and imaging use were collected. A cost-consequences analysis, undertaken from a healthcare provider perspective (hospital) estimated the incremental resource use, costs and effects of the Podiatry Triage Clinic relative to usual care over a 12-month period.

**Results:**

The Orthopaedic Surgeons and Podiatrist consulted with 72 and 212 new patients during the usual care and triage periods, respectively. The Podiatrist consulted with more new patients / session, mean (SD) of 3.6 (1.0) versus 0.7 (0.8), *p* < 0.001 and utilised less appointments / patient than the Orthopaedic Surgeons, mean (SD) of 1.3 (0.6) versus 1.9 (1.1), *p* < 0.001. The percentage of patients discharged without surgery was similar in the Podiatry Triage Clinic and usual care, 80.3% and 87.5% *p* = 0.135, respectively, but the surgical conversion rate was higher in the Podiatry Triage Clinic, 76.1% versus 12.5% *p* < 0.001. The total integrated appointment cost for the 12-month usual care period was $32,744, which represented a cost of $454.78 / patient. The total appointment and imaging cost during the triage period was $19,999, representing $94.34 / patient. Further analysis, suggests that the projected annual saving of integrating a Podiatry Triage Clinic versus an orthopaedic clinic alone is $50,441.

**Conclusions:**

The integration of a Podiatrist into an orthopaedic department significantly increases the number of patients seen, is cost-effective, improves the surgical conversion rate and improves the utilisation of Orthopaedic Surgeons.

## Background

Allied health-led triage clinics operate in both outpatient and emergency settings throughout Australia. These clinics frequently involve task substitution of an allied health professional for a medical practitioner, with the nature of work performed in these clinics varying from screening-only to assessment and implementation of treatment. The clinics are usually designed to identify patients unlikely to benefit from surgical intervention, and provide a non-surgical management plan. They are also used to detect patients who are likely to benefit from seeing a surgeon. The introduction of these clinics is often pursued by healthcare providers due to; (i) extended waiting periods for patients to access medical specialists and, (ii) the presumption that the clinics are both clinically- and cost-effective relative to usual care.

The clinical effectiveness of allied health professionals working in orthopaedic triage roles has been studied both in Australia, and internationally across a range of spinal [1], knee [2] and foot services [3]. Previous studies have primarily focused on patient-reported outcome measures and service efficiency [4–7], which indeed, support the use of allied health-led clinics. Whilst these outcome measures are important, ensuring that these clinics are also cost-effective is prudent given the increasing cost of providing health care. An evaluation of physiotherapy-led orthopaedic triage for spinal pain found these clinics to be economically favourable [4], however there is limited data to support the cost-effectiveness, or indeed the cost-consequences of implementing a podiatry-led orthopaedic triage clinic, for foot and ankle clinics.

The aim of this study therefore was to evaluate the cost-consequences of a podiatry-led triage clinic provided in an orthopaedic department relative to usual care to manage patients with non-urgent foot and ankle complaints.

## Methods

### Participants

All new patients (excluding trauma) seen for foot and ankle complaints in outpatients, by the Department of Orthopaedics and Trauma at The Queen Elizabeth Hospital between July 2014 and June 2016, were included in this analysis. New patients seen by Orthopaedic Surgeon-led clinics between July 2014 to June 2015 were considered ‘usual care’, while the patients seen in the Podiatry Triage Clinic between July 2015 to June 2016 were considered the ‘triage’ period. The majority of patients seen in the usual care and the triage periods had waited between 12 and 24 months for their initial appointment, but some had waited greater than 24 months. New and review patient appointments were given 30 min and 15 min, respectively during both periods. This study was approved by the Central Adelaide Local Health Network Human Research Ethics Committee, project number Q20160509.

### Patient triage

Prior to appointments being issued, patients were categorised by a Consultant Orthopaedic Surgeon via paper triage. Categories are based on the diagnosis and the description given by the patient’s general practitioner as, urgent (to be seen within 30 days), semi-urgent (to be seen within 90 days) or non-urgent (to be seen in within 2 years). Only non-urgent patients seen in clinic were included in the analysis. The common conditions seen as non-urgent are hallux valgus, hallux rigidus, hammertoes, Morton’s neuroma, plantar fasciopathy, midfoot osteoarthritis, Achilles tendinopathy and plantar plate pathology.

The Podiatry Triage Clinic was commissioned for six-hours each week to consult with non-urgent foot and ankle patients. The clinic was imbedded into existing orthopaedic clinics, utilising clinical nursing and administrative support services concurrently, thus enabling the Podiatrist to consult with other members of the orthopaedic team as needed. This approach also limited the additional costs of implementing the clinic, whereby the only costs incurred by the hospital were; (i) the additional administration to schedule patients and; (ii) the Podiatrist’s wages. Medical imaging was included in the economic analysis, although it is not a cost to the provider (hospital), it is a cost to the public health system.

The Podiatrist’s responsibilities were to evaluate new patients, provide a diagnosis and provide non-surgical treatments where indicated. Patients were discharged from the waiting list, with their consent if; (i) their symptoms resolved with non-surgical treatment, (ii) their condition was not amenable to surgical intervention, or (iii) they did not want surgery. Patients were referred to the Consultant Orthopaedic Surgeons if they failed non-surgical treatment or if they requested an appointment with a surgeon.

### Data collection

The data collected included patient demographics, the number of new and review appointments for each patient, medical imaging use, discharge rate and surgical conversion rate. Surgical conversion rate was collected from the commencement of the Podiatry Triage Clinic (July 2015) to December 2016. Orthopaedic patient costs were provided by the surgical directorate as an aggregated cost, while the Podiatrist and administration costs were estimated using hourly rates of local salaries, plus 20% on-costs. Medical imaging costs were calculated from the Medicare Benefits Schedule. Costs and benefits were not discounted because the analysis period was 12-months. Resource use was costed in 2016 Australian dollars. The data collected was de-identified prior to analysis.

### Data analysis

All data were checked for normality prior to inferential statistical analysis. The between group differences in age, number of appointments / patient and the number of new patients / session was analysed with the Mann-Whitney *U* test. The chi-squared test was used to analyse differences in gender, surgical conversion rate and the number of patients discharged without surgery. Descriptive summary statistics were estimated for outcomes and costs. The total costs and mean cost per patient with non-urgent foot and ankle complaints were calculated for the usual care and triage clinics. A cost-consequences analysis, from a provider (hospital) perspective, enables consideration of disaggregated outcomes and costs, allowing individual decision makers to make their own trade-offs between the costs and benefits of alternative models of service provision [8]. Statistical analyses were conducted using SPSS for Windows version 24.0 (SPSS, Inc., Chicago, IL) and Microsoft Excel (version 14.0).

## Results

### Participant characteristics

There were 72 and 212 non-urgent patients seen and provided with a definitive destination in the usual care and triage time periods, respectively. The median (interquartile range (IQR)) age of patients in the usual care and triage groups was 56.1 (20.4) years and 57.4 (18.7) years, respectively *p* = 0.901. There was no significant difference in gender between groups, with the usual care group having 59 / 72 (82.0%) women, while the triage period had 161 / 212 (75.9%) women, *p* = 0.418.

### Usual care outcomes

The Consultant Orthopaedic Surgeons conducted 110 sessions, 12 of the sessions were for the ‘complex foot and ankle clinic’ and involved the attendance of three consultants. There were 40 urgent, 95 semi-urgent and 72 non-urgent new patients seen during the pre-pilot period. There were 61 review appointments for non-urgent patients. The number of non-urgent patients discharged, without surgery, during this period was 63 resulting in a surgical conversion rate for non-urgent foot and ankle patients of 12.5%.

### Triage clinic outcomes

The Podiatrist consulted in 70 sessions, of the 255 new patients seen during this period, 212 have a definitive destination, while 43 were under review and were not included in the analysis. There were 66 review appointments during this period. Sixty-eight patients failed to attend appointments. There were 128 / 212 (60.3%) patients seen in the Podiatry Triage clinic who did not require an appointment with a Consultant Orthopaedic Surgeon and were discharged directly. Those referred to the surgeons had a surgical conversion rate of 76.1%. A summary of the usual care and triage clinic appointments is provided in Table 1.

**Table 1:** Summary of patients with non-urgent foot and ankle complaints managed in the usual care and triage clinics over the 12-month periods

	Usual care^a^	Triage^b^	*p* value
Total number of new patient appointments, n	72	212	
Total number of review patient appointments, n	61	66	
Total number of clinic sessions, n	110	70	
Age, years, median (IQR)	56.1 (20.4)	57.4 (18.7)	0.901^‡^
Gender, no. women (%)	59 (82.0)	161 (75.9)	0.418^‡‡^
Total new patients per session^c^, mean (SD)	0.7 (0.8)	3.6 (1.0)	< 0.001^‡^
Number of appointments per patient, mean (SD)	1.9 (1.1)	1.3 (0.6)	< 0.001^‡^
Number of patients discharged without surgery^d^, n (%)	63 / 72 (87.5)	139 / 173 (80.3)	0.135^‡‡^
Surgical conversion rate^e^	12.5%	76.1%	< 0.001^‡‡^

^a^outpatient clinic, patients with non-urgent foot and ankle complaints managed by Orthopaedic Surgeons and registrars (July 2014–June 2015);

^b^outpatient clinic, patients with non-urgent foot and ankle complaints managed by a Podiatrist (July 2015–June 2016);

^c^includes all new patients seen, including those currently under review in the triage clinic;

^d^includes patients discharged from triage clinic and those referred to Orthopaedics and discharged;

^e^surgical conversion rate is based on conversion from orthopaedic outpatient appointment to consent for surgery

^‡^ *p* calculated for differences between groups analysed with Mann-Whitney *U* test

^‡‡^ *p* calculated for differences between groups analysed with chi-squared test

Of the 84 patients referred to the Orthopaedic Surgeons from the Podiatry Triage Clinic between July 2015 and December 2016, 35 / 46 (76.1%) had consented to surgery, while 11 were discharged without surgery. Of the remaining patients, 12 are under review, 14 remain to be seen and 12 failed to attend their appointment with a Consultant Orthopaedic Surgeon. The patients seen by the Orthopaedic Surgeons, after seeing the Podiatrist, had a mean (SD) number of appointments of 1.3 (0.5).

There were no significant differences in the number of patients discharged, without surgical intervention, between the usual care and triage periods. With 63 / 72 (87.5%) patients discharged from the usual care and 139 / 173 (80.3%) patients discharged during the triage period, *p* = 0.135.

### Costs

The estimated costs of the usual care and triage clinics are summarised in Table 2. The average cost per orthopaedic clinic appointment (usual care) was $246.20. This includes wages (medical / nursing / administration / allied health) of $100.52, medical imaging ($38.67), pathology ($3.54), pharmacy ($7.78), ward supplies ($51.93), hotel ($6.88), non-clinical resources ($9.27), depreciation ($13.85) and on-costs ($13.76). The estimated total cost over 12-months for usual care patients was $32,744 ($454.78/ patient). On average, the triage clinic incurred direct costs of podiatry and administration wages at a rate of $39.34 / appointment ($51.59 / patient) and $42.75 / patient for medical imaging. Imaging use is detailed in Table 3. The estimated total appointment and imaging costs for the triage clinic were $19,999 ($94.34 / patient).

**Table 2:** Summary of the costs of managing patients with non-urgent foot and ankle complaints in the usual care and triage clinics over the 12-month periods

	Usual care^a^ (*n* = 133)			Triage^b^ (*n* = 278)		
	Unit cost	Number	Total cost	Unit cost	Number	Total cost
Total new and review appointments	$246.20	133	$32,744	$39.34 (wages)	278	$10,936
Imaging cost per patient			Included			$42.75
Total appointment cost			$32,744			$19,999
Mean cost per new patient			$454.78			$94.34

^a^outpatient clinic, patients with non-urgent foot and ankle complaints managed by Orthopaedic Surgeons (July 2014–June 2015);

^b^outpatient clinic, patients with non-urgent foot and ankle complaints managed by a Podiatrist (July 2015–June 2016)

**Table 3:** Summary of the utilisation of imaging in managing patients with non-urgent foot and ankle complaints in the usual care and triage clinics over the 12-month periods

Investigations	Usual care	Triage
X-Ray	57	98
Ultrasound	20	40
CT	1	3
MRI	3	3
Nuclear bone scan	3	1

*Abbreviations*: *X-Ray* Plain film radiograph, *CT* Computed tomography, *MRI* Magnetic resonance imaging

The estimated cost savings of integrating a Podiatrist into an orthopaedic department to manage 212 new patients with non-urgent foot and ankle complaints is $77,866 (($454.78 × 212 = $97,865) – $19,999). If the costs associated with additional appointments required for patients seen by the Orthopaedic Surgeons after triage (84 / 212) are also included, savings reduce to $50,441 ($77,866 – ($246.20 × 1.3 × 84 = $27,425)).

## Discussion

The findings from this study suggest the integration of a Podiatrist into an orthopaedic department to consult with non-urgent foot and ankle patients increases the number of new patients seen, improves surgical conversion rate and is more cost-effective than usual care. The Podiatrist effectively distils referrals from general practitioners and progresses only those likely to require operative intervention, improving the utilisation of specialist services in a tertiary care hospital. There are clear economic advantages of having an allied health practitioner perform a clinic triage for orthopaedic outpatients, with projected savings of more than $50,000 annually.

While 40% of the cohort required appointments with the Orthopaedic Surgeons following the Podiatry Triage Clinic, 60% were discharged directly. Moreover, the non-significant difference in the number of patients discharged, without surgery, during the usual care and triage periods also finds that over 80% of the non-urgent foot and ankle patients are managed non-surgically. The higher surgical conversion rate (76.1% versus 12.5%) also highlights the benefits of this clinic and improved use of a Consultant Orthopaedic Surgeons’ time. Furthermore, the surgeons also required fewer appointments with patients post-implementation of the Podiatry Triage Clinic (1.9 versus 1.3), further reducing costs. The discharge rate and the surgical conversion rate in this study are similar to other orthopaedic triage roles [1, 4, 6] suggesting that more than half of the non-urgent referrals made to orthopaedic departments can be managed by allied health professionals. The embedded nature of the triage clinic into an orthopaedic department ensures that suitable clinical governance was provided and collaborative care is readily available.

Despite orthopaedic clinics being led by foot and ankle Consultant Orthopaedic Surgeons, a variety of upper- and lower-limb conditions were managed in the ‘usual care’ orthopaedic outpatient clinics. Priority for outpatient appointments is given to the management of fractures, post-operative reviews and new urgent or semi-urgent cases, meaning the availability of non-urgent appointments is often delayed. Moreover, despite the increasing demands for specialist services at public hospitals in Australia, most surgical specialities spend more time consulting in the private as opposed to the public sector [9]. Orthopaedic Surgeons spend less than 30% of their time consulting in Australian public hospitals, if hospitals are to ensure that a surgeons’ limited time is appropriately utilised, then processes should be developed to ensure that the patients that are seen do require a surgical opinion. The introduction of clinics such as the Podiatry Triage Clinic, improves patient flow, reduces the number of surgeon-led appointments and progresses those more likely to require surgery. The Podiatrist consulted with a greater number of new, non-urgent foot and ankle complaints compared to the usual care period, (255 versus 72) respectively, and because the clinic was dedicated to non-urgent patients, was able to do so in 36% less sessions.

The integration of ‘gatekeeper’ services is an effective and cost-effective way of ensuring only those appropriate for surgery are consulted by surgeons. Orthopaedic Surgeons consult and manage a large volume of non-operative conditions [10], and may only operate on 30% of patients that attend their service [11]. Given the increased cost of a medical specialist’s wage, ensuring that patients see surgeons after they have exhausted non-operative measures will reduce costs and improve flow. Triage clinics also ensure non-operative management has been trialled for a period of time, prior to consultation with a surgeon, a factor that was previously found necessary from both Orthopaedic Surgeons and allied health professionals prior to surgical intervention [12]. The surgical management of the foot, in particular, has a high medical litigation rate [13] and therefore exhausting non-operative management prior to surgical intervention is perhaps even more important.

This economic analysis should be considered in light of some limitations. Firstly, a provider perspective was taken, which looked at appointments and surgical conversion rate, but did not investigate other outcomes such as patient-reported outcome measures. Furthermore, patient-level costs were unavailable and therefore estimates are based on average costs only. Costs beyond the current analysis e.g. additional treatments such as medicines, external appointments and orthoses / braces, and productivity costs are also not included. Secondly, the analysis also does not take into account time spent by the Podiatrist outside of the clinical time e.g. performing research, administration. Thirdly, patients were not randomly assigned to usual care or the triage clinic. Therefore, the observed differences could be influenced by other unmeasured variables. Fourthly, the costs of the usual care and triage clinics are estimated using data from one hospital in South Australia and may not be generalisable to other jurisdictions with different funding models. Finally, it is unknown if the patients discharged from the waiting list will return to this list in the future.

It should be noted that while triage clinics are cost-effective, the cost of introducing these clinics is an additional expense to usual care for health networks, but usual care for the majority of non-urgent foot and ankle complaints is to remain on a waiting list, often for years [14]. This clinic can identify those requiring surgery and expediting their care, whilst also providing non-surgical advice to those not requiring surgery. Indeed, this is an effective and economically prudent form of assessment, but the economic benefits are realised when the addition of a triage clinic is married with the reduction of a medical consultants’ clinic.

## Conclusion

The findings from this study suggest the integration of a Podiatrist into an orthopaedic department is cost-saving and improves the use of an Orthopaedic Surgeons’ time and patient flow. As the cost of providing health care increases, the introduction of such roles in other departments may be an appropriate and cost-effective use of qualified allied health professionals.

## Abbreviations

IQR: Interquartile range
SD: standard deviation
SPSS: Statistical Package for the Social Sciences
